# Biaxial Flexural Strength of Printed Splint Materials

**DOI:** 10.3390/ma17051112

**Published:** 2024-02-28

**Authors:** Johann Wulff, Angelika Rauch, Michael Benno Schmidt, Martin Rosentritt

**Affiliations:** Department of Prosthetic Dentistry, UKR University Hospital Regensburg, 93042 Regensburg, Germany; johann-philip.wulff@ukr.de (J.W.); angelika.rauch@ukr.de (A.R.); michael3.schmidt@ukr.de (M.B.S.)

**Keywords:** 3D printing, oral splint, biaxial flexural strength, TMD, PMMA, DLP

## Abstract

One therapeutical alternative in the treatment of functional disorders is the use of printed oral splints. The mechanical properties of these materials are highly essential to their clinical effectiveness, and their performance may vary depending on factors such as cleaning, post-polymerization, or their orientation during construction. The objective of this in vitro investigation is to evaluate the effectiveness of the selected materials in terms of their biaxial flexural strength in relation to the criteria listed above. Splint materials were used in the printing of 720 discs. The printing process was carried out in different orientations in relation to the building platform. Either an automatic or manual cleaning process was performed on the samples. For post-polymerization, either an LED or Xenon light was utilized. A piston-on-three-ball test was used to measure the biaxial flexural strength (BFS) of the materials after they were stored in water for either 24 h or 60 days. The homogeneity of the data was controlled by employing the Levene method, and the differences between the groups were analyzed using the ANOVA and Bonferroni methods. After being stored for twenty-four hours, the mean BFS ranged anywhere from 79 MPa to 157 MPa. Following a period of sixty hours, the BFS exhibited a substantial drop and revealed values that ranged from 72 to 127 MPa. There was no significant difference that could be identified between the materials or between the various cleaning processes. The results of post-polymerization showed that the LED light produced higher means than the Xenon light did. In terms of position, the mean values varied greatly, with 0°’s mean value being 101 MPa, 45°’s mean value being 102 MPa, and 90°’s mean value being 115 MPa. The use of a build orientation of 90° and post-polymerization with LED light resulted in significantly increased biaxial flexural strength. According to this study, this design should be implemented in order to ensure that splint materials have the highest possible strength.

## 1. Introduction

Temporomandibular disorders, also known as TMD, is a broad term that encompasses a variety of discomfort and dysfunctional conditions that affect the temporomandibular joints (TMJs) and the masticatory muscles [1]. The most notable characteristics of this condition are the presence of pain in the face and preauricular region, restrictions on jaw mobility, and noise from the temporomandibular joints when moved. The patient’s quality of life may be negatively impacted by TMD, which have been linked to a number of psychological conditions, including depression, reduced general health, and several other grievances. Noninvasive therapies, such as behavior therapy, medication, physical therapy, and occlusal appliances, have been shown to be effective in treating individuals with temporomandibular disorder [1,2,3].

When it comes to the treatment of temporomandibular disorders and parafunctions like bruxism, oral splints are often utilized among dental professionals [4]. Bruxism is defined as repetitive muscle activity, which involves clenching or grinding of the teeth and/or thrusting of the jaw. It can be classified as either sleep bruxism or awake bruxism [4]. The utilization of oral splints has been defined as a viable treatment approach for the management of bruxism [5]. One of the objectives of oral splints is to provide uniform, balanced, and unhindered occlusal contacts. These splints are meant to accomplish this objective without permanently affecting the position of the mandible or the patient’s occlusion. Furthermore, a splint that is well-designed will encourage a harmonious relationship between the structures in the stomatognathic system [6,7]. Customization of oral splints could also ensure better comfort and herewith a better compliance [8].

For the most part, cast methacrylates or deep-drawn thermoplastics are utilized in the construction of adjustable oral splints [9]. These are part of a well-established analog workflow consisting in taking alginate impressions of the upper and lower jaw, bite registration, cast fabrication, splint wax-up, and using powder-liquid mixtures to produce the splints. In spite of this, the process for digital production has shown consistent development over the past few years. Computerized optical scans of the upper and lower jaw, digital bite registration, computer-aided design (CAD), and computer-aided manufacturing (CAM) are all components of the digital workflow that is now utilized in the process of fabricating splints [7,10]. The computer-aided manufacturing (CAM) process can be carried out using either subtractive techniques, such as milling of industrially produced Polymethylmethacrylate (PMMA) blanks, or additive techniques, such as stereolithography, digital light processing, or material jetting [7,11]. Benli et al. stated that it appears that it is possible to achieve mechanically and chemically acceptable qualities with PMMA-based oral splint materials by employing both conventional and digital production processes. Based on this review, it seems that additively manufactured alternatives show less attractive mechanical and chemical properties as their milled counterparts [12]. In spite of this, additive manufacturing, often known as 3D printing, is an acceptable alternative for the rapid creation of splints in the event that fractures or loss occur. There are digital light processing (DLP) VAT 3D printing systems that make use of liquid photopolymer resins [10,13]. It was noted by Alharbi et al. that in order to maximize the efficiency of the process, it is necessary to properly coordinate the printing techniques and the materials [14]. This can be accomplished, for example, by taking into account the type of printer and its speed in conjunction with the appropriate viscosity of the resin that is being used [15]. In order to remove any uncured monomer, the splints need to be washed with a solvent (such as 2-propanol) immediately after the printing process has been completed [16]. Despite the fact that conversion is partially restricted [17,18], additive manufacturing makes it possible to fabricate splints with adequate accuracy [19,20,21]. Immediately following the cleaning process, the splints are post-polymerized with the assistance of external light sources, such as LED or Xenon devices, in order to ensure that the final polymerization is sufficient. As a result of the major differences that exist across the various printing techniques, the effects of the material, cleaning, and polymerization are of particular significance.

Park et al. [22] stated that printed splints are more flexible than hand-cast or milled solutions, while yet maintaining an appropriate level of mechanical strength. It would appear that the thickness and orientation of the individual layers during the manufacturing process have an effect on the mechanical properties of the splints [23,24,25,26]. The orientation of the layers within the product is something that needs to be taken into consideration due to the fact that it has an effect on the material’s strength [14,27].

When constructing an object, it is important to keep in mind the mechanical properties of the materials used to build the occlusal splints. It is possible for the splint to become deformed, fracture, or perform less effectively if it is insufficiently strong or has an incorrect design. Due to the fact that bite forces can produce values of up to 999.3 N and that it is typical for splints to be inserted and removed at a high frequency during clinical service, the flexural strength of splint materials is of great significance [28,29,30,31]. According to [23] and DIN EN ISO 20795-1:2013-06, tests such as the three-point bending test and the biaxial flexural strength test can be utilized to determine whether or not the materials in question satisfy the current standards. These tests also provide the manufacturer with the threshold values that are relevant to the situation. One of the benefits of the biaxial test in comparison to the conventional three-point bend test is that it eliminates the possibility of edge effects. In addition to ensuring that the stress field is homogeneous across a wide portion of the sample, it is also possible to avoid the influence of sample orientation. In order to reduce the effects of friction, movable supports or additional intermediary soft layers on the surfaces of the samples are typically utilized. Long-term effects, such as those brought on by a damp environment, should be taken into account in order to imitate clinical performance.

Hence, the objective of this study is to examine the strength of 3D printed splint materials, while simultaneously evaluating the efficacy of automated and human post-processing methods and considering the influence of pre-processing parameters. This in vitro research aims to determine whether printed splint materials´ biaxial flexural strength would be unaffected by build orientation (0°, 45°, 90°), cleaning (manually, automatically), post-polymerization (LED, Xenon), or storage (24 h, 60 days).

## 2. Materials and Methods

### 2.1. Printing

**Figure 1:**
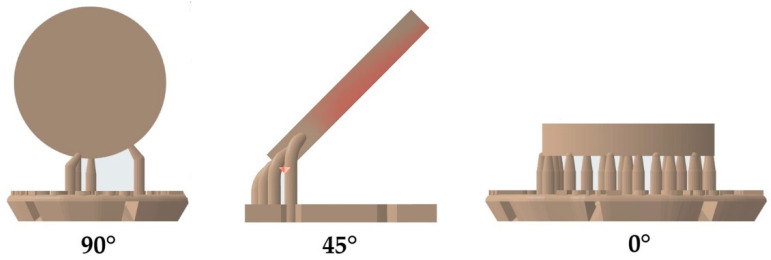
Sample design: orientation to building platform.

Using a P30+ DLP-printer (Straumann, Basel, Switzerland), samples were fabricated from two different splint materials (M1: Luxaprint OrthoPlus, DMG, Hamburg, Germany; M2: V-Print Splint, VOCO, Cuxhaven, Germany) (Table 1). The samples had a diameter of 16 mm and a height of 2 mm. The three-dimensional printing process was carried out with the object oriented at 90 degrees, 45 degrees, or 0 degrees angles to the building platform (Figure 1). The thickness of the layers was 100 µm, and the design included supporting structures. A total of 720 samples were printed, with 15 samples being printed for each group and test.

### 2.2. Cleaning

Following the completion of the printing process, the samples were cleaned using either an automated (AUTO: P Wash, Straumann, Basel, Switzerland) or a manual (MAN: VOCO Pre-/Main-Clean protocol, VOCO, Cuxhaven, Germany) cleaning procedure. As a cleaning agent, isopropanol was utilized in each of the regimens.

### 2.3. Post-Polymerization

After the samples were cleaned, these were subjected to a post-polymerization process. External curing devices using either LED (LED: P Cure, Straumann, Basel, Switzerland) or Xenon lights (XEN: Otoflash N171, Ernst Hinrichs Dental, Goslar, Germany) were used for this purpose.

### 2.4. Processing

After removing the supporting structures using rotating instruments, the samples were ground using silicon carbide paper grit, P600/1200 (Paper SiC P600/1200; Struers GmbH, Willich, Germany), to their final dimensions. Following that, the samples underwent pre-polishing with pumice flower and were subsequently high gloss polished (Universal Polishing Paste; Ivoclar Vivadent, Schaan, Liechtenstein). The samples were cleaned in an ultrasonic bath for two minutes to remove any leftover debris, rinsed with distilled water, and then dried with compressed air.

### 2.5. Storage

Samples were kept for either 24 h (*n* = 360) or 60 days (*n* = 360) in distilled water at 37 °C.

### 2.6. Testing

In order to evaluate biaxial flexural strength (BFS), a piston-on-three-ball test based on ISO 6872 was performed using a universal testing machine (Z2.0, Zwick/Roell, Ulm, Germany; THS1620, Grip-Engineering Thümler, Nürnberg, Germany). The samples were positioned on a ring-like bearing, which was made up of three stainless steel spheres with a diameter of three millimeters each. These spheres were arranged in the shape of an equilateral triangle at a 120-degree angle having a diameter of ten millimeters. The samples were positioned in the middle of the bearing. In order to ensure that the contact force was distributed uniformly, a polyethylene film with a thickness of 0.05 mm (1-7090, neoLab Migge, Heidelberg, Germany) was positioned between the sample and the piston as well as between the sample and the bearing. A preload of 0.5 N was applied to the samples. The load was applied at a rate of one millimeter per minute (1 mm/min) by the piston, which had a diameter of 1.6 mm.

The fracture force was measured, and the BFS was obtained using the following equation:

σ = −0.2387P(X − Y)/d^2^ (1)

Legend:

σ = biaxial flexural strength (MPa);
P = fracture force (N);
d = sample thickness at fracture origin (mm).

The variables X and Y were determined as follows:

X = (1 + v) ln(r_2_/r_3_)^2^ + [(1 − v)/2] r_2_^2^/r_3_ (2)

Y = (1 + v) [1 + ln(r_1_/r_3_)^2^] + (1 − v) (r_1_/r_3_)^2^ (3)

Legend:

v = Poisson’s ratio (0.3) [32];
r_1_ = radius of the supporting bearing;
r_2_ = radius of the loaded area;
r_3_ = radius of the sample.

### 2.7. Statistics

Statistics were performed (SPSS 26.0, IBM, Armonk, NY, USA). Data were controlled for homogeneity using a Levene test, and differences between the groups were analyzed (one-way ANOVA, Bonferroni) at a level of significance of α < 0.05. Intermediate subject effects were calculated.

## 3. Results

### 3.1. Build Orientation

Regarding orientation, the mean values ranged between 72 MPa and 141 MPa for 0°, 73 MPa and 122 MPa for 45°, and 83 MPa and 146 MPa for 90°. A significant effect of build orientation on the BFS of printed 3D materials could be observed (mean_0_ 101 MPa, mean_45_ 102 MPa, and mean_90_ 115 MPa; *p* < 0.001)

### 3.2. Cleaning

No significant differences could be found among the various cleaning procedures utilized (*p* = 0.138).

### 3.3. Post-Polymerization

The mean BFS after post-polymerization with LED light varied between 85 MPa and 158 MPa. Post-polymerization with Xenon light showed values ranging from 72 MPa to 128 MPa. A significant influence of the post-polymerization process could be observed (mean_LED_ 115 MPa, mean_XEN_ 95 MPa; *p* < 0.001)

### 3.4. Storage

**Figure 2:**
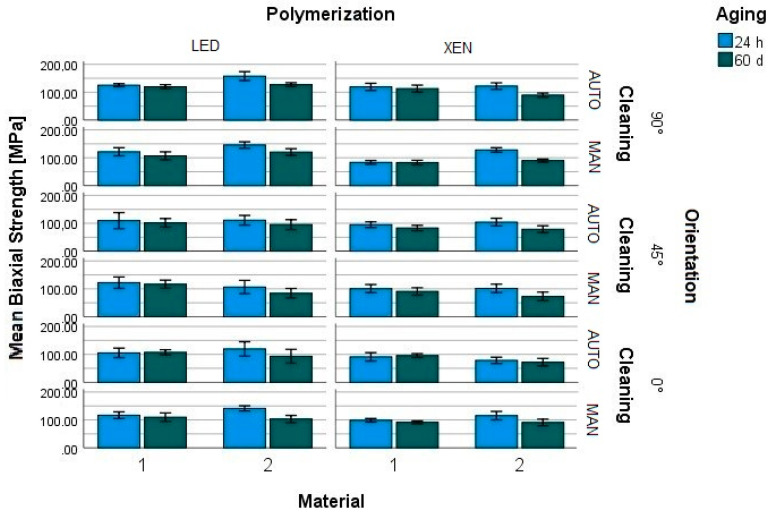
Mean biaxial flexural strength of splint materials (M1, M2) depending on polymerization (LED, XEN), cleaning (AUTO, MAN), storage in water (24 h, 60 days), and printing orientation (90°, 45°, 0°) to the building platform.

The mean BFS after 24 h of storage varied between 78 MPa and 158 MPa. After 60 h, the BFS revealed values ranging from 71 MPa to 128 MPa (Figure 2). A significant influence of storage could be observed (mean_24_ 113 MPa, mean_60_ 97 MPa; *p* < 0.001).

### 3.5. Statistics

The Levene test revealed that the error variance of the dependent variable was different across groups (*p* < 0.001), thus a significance level of 0.01 was set. Intermediate subject effects were determined for the combination of material and position/aging (*p* < 0.001), position and cleaning/polymerization (*p* < 0.001), material and position and cleaning (*p* < 0.001), material and position and polymerization (*p* < 0.001), and material and cleaning and polymerization (*p* < 0.001). All other combinations revealed no intermediate effects (*p* ≥ 0.012) (Table 2).

## 4. Discussion

In this in vitro study, the hypothesis was that the biaxial flexural strength of the printed splint materials would not be impacted by build orientation (0°, 45°, 90°), cleaning (MAN, AUTO), post-polymerization (LED, XEN), or storage (24 h, 60 days). However, it was not possible to confirm this hypothesis. Biaxial flexural strength was significantly affected by the build orientation as well as by the type of polymerization and water storage that was applied. Together, these factors had a large impact on the biaxial flexural strength of the materials.

In spite of the fact that there is a substantial body of research accessible on the subject of the precision of printed dental devices, there is a limited quantity of evidence concerning the mechanical qualities of dental products that have been manufactured using 3D printing. Comparative studies have been conducted in the past to examine the differences and similarities between pressed, milled, and 3D printed splint materials. The results demonstrated that after being aged, resins that were created using 3D printing have lower values for both flexural strength and hardness. [13].

### 4.1. Build Orientation

In the course of this research, the samples that were printed at a 90-degree angle yielded the greatest BFS values. On the other hand, when a lower printing angle was used, it was discovered that the flexural strength of the material decreased. Previous investigation on build orientation with AM resins have shown that the orientation of the individual layers during the printing process has an effect on the mechanical characteristics of the devices that are printed [23,24]. Earlier studies [33,34] have described an anisotropy of additive materials that is brought about by the orientation during the printing process. When employing a DLP printer, a collection of micro-mirrors is exposed to ultraviolet light, which simultaneously polymerizes a layer that is composed of a large number of voxels. The voxels are polymerized vertically without any gaps between them, layer by layer, which results in the generation of columns. Nevertheless, a reduced degree of polymerization is produced in these sectors as a consequence of the presence of thin interstitial or shadow zones that split the voxels from one another in the lateral direction. It is possible that a structural weakness is present since these regions coincide with the perimeters of each and every micro-mirror [33,35,36]. The conversion in these areas takes place first during the post curing phase while using external curing devices [33].

### 4.2. Cleaning

It did not appear that the cleaning technique that was used had a significant effect on the flexural strength of the 3D printed materials that were put through extensive testing. The flexural strength of materials has been shown to decrease when the cleaning period is dramatically increased from five minutes to twelve hours, as noted by Xu et al. [37]. Hwankgbo et al. were also able to determine that the length of time used for cleaning 3D printed devices has a significant influence on their mechanical properties, including their flexural strength and flexural modulus [38]. When it comes to the mechanical characteristics of printed devices, it would appear that time is of more significance than the employment of either an automated or manual method or both. When analyzing other characteristics of splint materials, such as cytotoxicity, it was possible to see significant effects of the cleaning technique that was applied when conducting the tests [39]. Studies have demonstrated that the use of automatic cleaning of the samples, as opposed to manual cleaning, results in a reduction in the cytotoxicity of the tested materials [39].

### 4.3. Post-Polymerization

During the course of this experiment, post-curing with LED lighting led to increased values for biaxial flexural strength. This could be due to a decrease in the quantity of residual monomers or an increase in the conversion of double bonds, both of which are viable explanations for this phenomenon. It is possible that the higher conversion could be attributed to a variable energy input or the applied wavelength from the polymerization devices, which adjusts to the absorption spectrum of the photo initiator. When compared to Xenon curing, LED curing appears to be more effective and dependable under the conditions that were utilized. These findings are supported by a previous study that demonstrated that the combination of vacuum curing and the heating capability of LED light may increase the conversion of specimens and decrease the presence of oxygen inhibition on the outermost layers [40]. The process of polymerizing methacrylate resins through chain reactions is an intricate one that is affected by a variety of factors, including the composition of the materials, the curing equipment that is utilized, and the ambient conditions that are present in the surrounding environment [41]. The degree of conversion, hardness, and biocompatibility of resins that are used in 3D printing can be improved by extending the post-polymerization stage to a longer period of time [42,43]. There is a possibility that the degree of conversion could be increased through the effective combination of heat and light during the post-polymerization process [18].

### 4.4. Storage

Water storage had a crucial effect on BFS, resulting in a reduction of mean values ranging from 1% to 30%. One possible explanation for the decline in the mean BFS following aging could be the considerable water uptake (−27.7 μg/mm^3^) or the solubility (<0.1 μg/mm^3^) of the material. Various research groups have already established that the behavior of dental resins is influenced by the corrosive influence of water and/or by the periodic masticatory stresses that are applied to the material. Elution, deterioration, relaxation, accelerated crack growth, visco-elastic effects, and decreased wear resistance are all potential outcomes that might be brought about by these causes [31,44,45,46].

Despite the fact that previous studies have demonstrated that the mechanical qualities of 3D printed materials are often inferior to those of milled materials, the technology of additive manufacturing represents a promising field for dentistry [47]. Within the scope of future studies, it is possible to incorporate a more extensive range of materials into a clinical environment (in vivo) in order to investigate the durability and clinical acceptance of these materials. Printer settings regarding different build orientations and layer thickness could be addressed as a future topic to achieve optimal performance of the materials available for additive splint manufacturing.

## 5. Conclusions

- Samples printed at a build angle of 90° showed the highest biaxial flexural strength values in comparison with those printed at either 0° or 45°.
- Samples cured with an LED device showed higher biaxial strength values than those cured with Xenon light.
- The aging of the samples significantly decreased the mechanical properties of the materials.

Therefore, in order to achieve the best results possible in regard to the mechanical properties of 3D printed objects in terms of their biaxial strength, it is necessary to carefully match the post-processing of printed objects. Therefore, in light of the findings of the current research, it is recommended that a design that incorporates a construction orientation of 90 degrees and post-polymerization with LED light be applied in order to guarantee that splint materials possess the highest possible strength.

## Figures and Tables

**Table 1:** Study design, materials and devices.

	Abbr. Text	Device/Material Protocol	Manufacturer LOT
**Printer**		P30+ (digital light processing)	Straumann, Basel, Switzerland
**Orientation**	**0°**		
	**45°**		
	**90°**		
**Cleaning**	**AUTO**	P Wash (isopropanol): pre-cleaning 3:10 min, cleaning 2:20 min, drying 1:30 min	Straumann, Basel, Switzerland
	**MAN**	Pre-/Main-Clean (isopropanol): pre-cleaning 3:00 min, ultrasonic: 2:00 min, air-drying: 1:00 min	VOCO, Cuxhaven, Germany
**Post-polymerization**	**LED**	P Cure: LED, 10 min, vacuum, UV-A: 400–315 nm; UV-B: 315–280 nm, heating	Straumann, Basel, Switzerland
	**XEN**	Otoflash G171: 2 × 2000 Xenon flashes, 280–700 nm, maximum between 400–500 nm	NK-OPTIK, Baierbrunn, Germany
**Materials**	**M1**	Luxaprint OrthoPlus: >90% bisphenol A dimethacrylate, 385/405 nm, flexural strength ≥ 70 MPa, flexural modulus ≥ 1 GPa, Shore D ≥ 60	DMG, Hamburg, Germany LOT 218479
	**M2**	V-Print Splint: acrylate, Bis-EMA, TEGDMA, hydroxypropyl methacrylate, butylated hydroxytoluene, diphenyl(2,4,6-trimethylbenzoyl) phosphine oxide, 385 nm, flexural strength 75 MPa, flexural modulus ≥ 2.1 GPa, water uptake 27.7 μg/mm^3^, solubility < 0.1 μg/mm^3^	VOCO, Cuxhaven, Germany LOT 2023138

**Table 2:** Intermediate subject effects (significance of α = 0.01; R^2^ = 0.647; grey: significant effects).

	F	*p*-Value
material	2.668	0.103
orientation	100.342	<0.001
cleaning	0.984	0.321
polymerization	356.934	<0.001
aging	228.539	<0.001
material × position	36.020	<0.001
material × cleaning	8.985	0.003
material × polymerization	2.966	0.085
material × aging	92.072	<0.001
position × cleaning	46.331	<0.001
position × polymerization	6.678	0.001
position × aging	2.904	0.056
cleaning × polymerization	0.542	0.462
cleaning × aging	5.412	0.020
polymerization × aging	0.124	0.724
material × position × cleaning	28.301	<0.001
material × position × polymerization	10.567	<0.001
material × position × aging	2.274	0.104
material × cleaning × polymerization	25.823	<0.001
material × cleaning × aging	0.650	0.420
material × polymerization × aging	0.026	0.872
position × cleaning × polymerization	1.688	0.186
position × cleaning × aging	3.345	0.036
position × polymerization × aging	4.367	0.013
cleaning × polymerization × aging	0.000	0.988
material × position × cleaning × polymerization	3.557	0.029
material × position × cleaning × aging	0.318	0.727
material × position × polymerization × aging	4.161	0.016
material × cleaning × polymerization × aging	0.945	0.331
position × cleaning × polymerization × aging	0.222	0.801
material × position × cleaning × polymerization × aging	1.055	0.349

## Data Availability

The data presented in this study are available on request from the corresponding author and are available in Supplementary Materials.

## Supplementary Materials

The following supporting information can be downloaded at: https://www.mdpi.com/article/10.3390/ma17051112/s1, Table S1. Biaxial flexural strength (BFS in MPa, mean, standard deviation [SD]) of the materials after different orientation to the building platform, cleaning, post-polymerization, and storage time in water.
