# Supplementary material for: Biaxial Flexural Strength of Printed Splint Materials
Source: Materials (Basel). 2024 Feb 28;17(5):1112. doi: 10.3390/ma17051112 (PMC10934272; doi:10.3390/ma17051112)
Supplement: Supplementary file 1 [file materials-17-01112-s001.zip › materials-2760887-supplementary.pdf]

Table S1. Biaxial flexural strength (BFS in MPa, mean, standard deviation [SD]) of the materials after different orientation to the building platform, cleaning, post-polymerization, and storage time in water.

Orientation to building platform	Material	Cleaning	Post-polymerization	storage time (water 37°C)	BAF	
					mean	SD
90°	M1	AUTO	LED	24 h	125.2	5.3
				60 d	119.7	7.5
			XEN	24 h	119.3	12.8
				60 d	112.8	12.6
		MAN	LED	24 h	121.3	14.9
				60 d	106.9	14.2
			XEN	24 h	83.3	6.9
				60 d	82.8	8.3
	M2	AUTO	LED	24 h	157.5	16.2
				60 d	127.1	6.9
			XEN	24 h	122.1	11.6
				60 d	89.5	7.5
		MAN	LED	24 h	145.8	11.5
				60 d	120.3	11.9
			XEN	24 h	127.9	8.0
				60 d	89.8	5.8
45°	M1	AUTO	LED	24 h	109.6	28.8
				60 d	101.7	15.3
			XEN	24 h	95.1	10.1
				60 d	83.0	9.6
		MAN	LED	24 h	122.2	20.4
				60 d	116.5	14.6
			XEN	24 h	100.6	14.8
				60 d	90.7	13.3
	M2	AUTO	LED	24 h	111.1	17.3
				60 d	95.3	17.8
			XEN	24 h	104.2	13.8
				60 d	78.6	12.5
		MAN	LED	24 h	106.5	23.9
				60 d	84.4	17.0
			XEN	24 h	101.4	15.2
				60 d	72.7	15.4
0°	M1	AUTO	LED	24 h	105.4	16.9
				60 d	107.4	9.1
			XEN	24 h	91.1	15.0
				60 d	95.5	6.9
		MAN	LED	24 h	116.7	12.1
				60 d	109.6	15.4
			XEN	24 h	98.5	6.4
				60 d	91.3	5.1
	M2	AUTO	LED	24 h	119.4	25.9

			60 d	93.2	24.8
		XEN	24 h	78.6	11.1
			60 d	71.9	13.5
	MAN	LED	24 h	141.3	9.3
			60 d	102.9	13.0
		XEN	24 h	115.2	14.9
			60 d	90.9	11.7
